# A severe coronavirus disease 2019 patient with high-risk predisposing factors died from massive gastrointestinal bleeding: a case report

**DOI:** 10.1186/s12876-020-01458-x

**Published:** 2020-09-29

**Authors:** Taojiang Chen, Qin Yang, Hongyu Duan

**Affiliations:** 1 Department of Pediatric Cardiology, West China Second University Hospital, Sichuan University, Chengdu, Sichuan China; 2 Key Laboratory of Birth Defects and Related Diseases of Women and Children (Sichuan University), Ministry of Education Chengdu, Chengdu, Sichuan China; 3 Department of Pediatric Cardiovascular Disease, West China Second University Hospital, Sichuan University, No. 20, Section 3, RenminNanLu Road, Chengdu, Sichuan 610041 China; 4 Department of Critical Care Medicine, Chengdu Fifth People’s Hospital, Chengdu, Sichuan China; 5 Cancer Center, Union Hospital, Tongji Medical College, Huazhong University of Science and Technology, Wuhan, Hubei China

**Keywords:** SARS-CoV-2, Coronavirus disease 2019, High-risk predisposing factors, Gastrointestinal bleeding

## Abstract

**Background:**

SARS-CoV-2 is highly infectious and has been a significant public health threat. Despite typical manifestations of illness are dominated by respiratory symptom, some patients have concurrent gastrointestinal manifestations, including nausea, diarrhea, and vomiting. Massive gastrointestinal bleeding, however, has rarely been reported.

**Case presentation:**

We herein described a case of severe SARS-CoV-2 infected patient with several risk factors for poor prognosis, including male, hypertension, old age, mixed bacterial infection and multilobular infiltration on radiological imaging. After improvement of respiratory status, the onset of gastrointestinal bleeding occurred, probably resulting from direct viral invasion as evidenced by the positive findings for SARS-CoV-2 in the repeat stool specimens. Although aggressive resuscitation was administered, hematochezia was uncontrolled. The patient rapidly deteriorated, suffered from cardiac arrest, and expired.

**Conclusions:**

Digestive symptoms could be severe in SARS-CoV-2 infected patients, especially for the high-risk individuals with predisposing conditions. A more thorough protocol for preventing cross-infection through faecal-oral transmission should be implemented in the process of patient care and infection control.

## Background

Since December 2019, SARS-CoV-2 has become a significant public health threat around the world. SARS-CoV-2 is an enveloped, non-segmented, positive-sense RNA virus belonging to the beta genus of the coronavirus family. It has been shown to possess a strong capability to infect humans through a binding of the viral S protein to angiotensin converting enzyme II (ACE2) on human cells [1]. Although most patients have mild symptoms and good prognosis, several predisposing factors including male, hypertension, old age, mixed bacterial infection and multilobular infiltration on radiological imaging have been proposed to be associated with poor outcomes [2]. Therefore, for the vulnerable individuals with aforementioned risk factors, prompt and effective management to interrupt disease progression is of crucial importance in reducing complications and mortality. Relevant experience, however, appears to be very limited in our knowledge.

Additionally, despite the clinical manifestations of coronavirus disease 2019 (COVID-19) are dominated by respiratory symptoms, evidences from recent studies have suggested that SARS-CoV-2 has the ability to actively infect and replicate in the gastrointestinal tract [3]. Gastrointestinal manifestations including nausea, diarrhea, and vomiting has been observed in cases with SARS-CoV-2 infection. Referring to massive gastrointestinal bleeding (GIB), rare data is available in the relevant literature. It has been reported that most of the patients with SARS-CoV-2 with GIB responded well to conservative management with careful monitoring, transfusion of red blood cells as needed, and medical therapy. The severity of their respiratory failure was far more responsible for the mortality risk than that of GIB. The deaths directly related to GIB was uncommon [4–7].

Herein, we described an old-aged COVID-19 patient with multiple risk factors for severe disease and ultimately died from massive GIB at Wuhan Union Hospital. We aimed to share some experience for prevention of disease progression in COVID-19 patients with defined risk factors, in order to improve the outcomes in such patients. We also hoped our findings could shed some light on gastrointestinal aspects of the disease.

## Case presentation

A 84-year-old man presented to a fever clinic of his living community in Wuhan, with a 15-day history of fever, cough, chest discomfort and fatigue. Except for histories of hypertension effectively controlled by oral intake of nifedipine and candesartan and surgery for disc herniation, the patient was a nonsmoker without other previous chronic underlying diseases. After 4 days of the antibiotic treatment (moxifloxacin, 0.4 g per day), his cough and chest discomfort worsened, but the fever was resolved. His two separate oropharyngeal swabs at>24 h intervals were both positive detected by a real-time reverse-transcriptase-polymerase chain reaction (rRT-PCR) assay for SARS-CoV-2. Finally, he was confirmed with the diagnosis of COVID-19 pneumonia, and transferred to Wuhan Union Hospital. On arrival, he was afebrile, no tachypnea, no tachycardia, no hypotension (blood pressure, BP: 120/75 mmHg), and no hypoxia (SpO2: 95% on room air). Other than rough respiratory sounds, the rest of his physical examination was unremarkable.

**Fig. 1:**
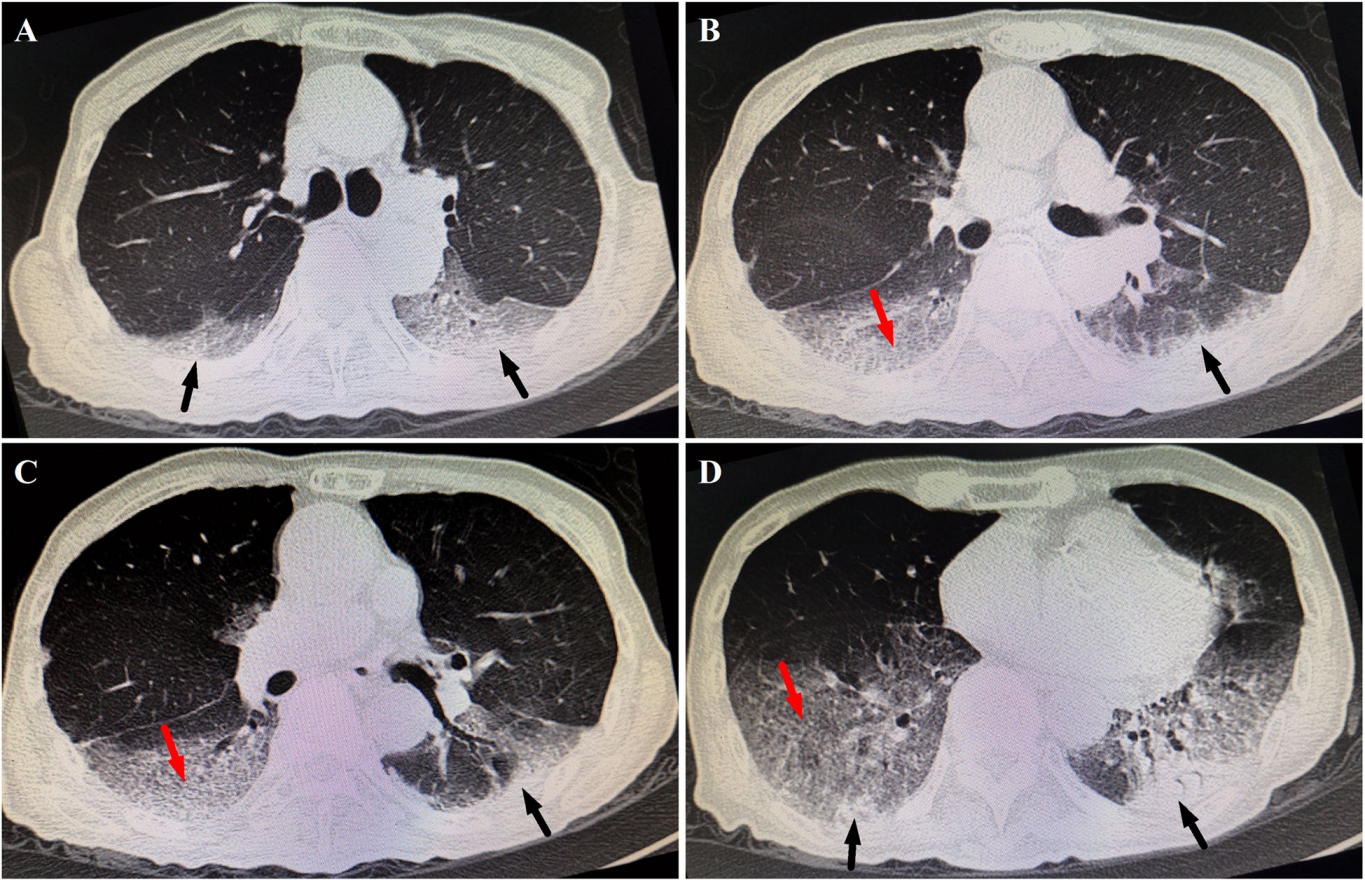
Selected images from chest computed tomography scans at different levels on hospital day 5. Subpleural multiple consolidations (black arrows) associated with smooth interlobular and intralobular septal thickening representing crazy paving pattern (red arrows) mainly in lower lobes in both lungs

Initial laboratory investigations revealed increased leukocytes (15,400/μm) with 91% neutrophils and 3.8% lymphocytes, a normal hemoglobin (15.0 g/dL), normal platelets (23.0 × 10^4^/μL), and elevated C-reactive protein (CRP) (1.62 mg/dL). Occult blood and rRT-PCR for SARS-CoV-2 in stool specimens were both negative. Liver/renal function, myocardial troponin I, electrolytes, total cholesterol, blood gas, and glucose were also normal. The patient received antiviral treatment (arbidol, orally 200 mg thrice daily), but worsening respiratory distress with percutaneous oxygen saturation of 89% even via a face mask was noted on hospital day 5. Intermittent fevers and rales in both of lung areas were also found. Laboratory results reflected increased leukocyte (21,200/μm), procalcitonin (PCT) (1.2 ng/mL), and CRP (6.41 mg/mL). An enhanced chest computed tomography (CT) demonstrated multiple patchy ground-glass opacities, and consolidations of the middle and inferior lobes in both lungs, associated with crazy paving pattern (Fig. 1). According to the severity of hypoxemia, the case was defined as the severe SARS-CoV-2 infected pneumonia [8]. High flow nasal oxygen therapy (HFNO), methylprednisolone (intravenously 80 mg per day for 3 consecutive days), piperacillin-tazobactam, intravenous immunoglobulin (IVIG), and Chinese herbs were given, combined with omeprazole and hydrotalcite for prevention of stress-induced GIB.

**Fig. 2:**
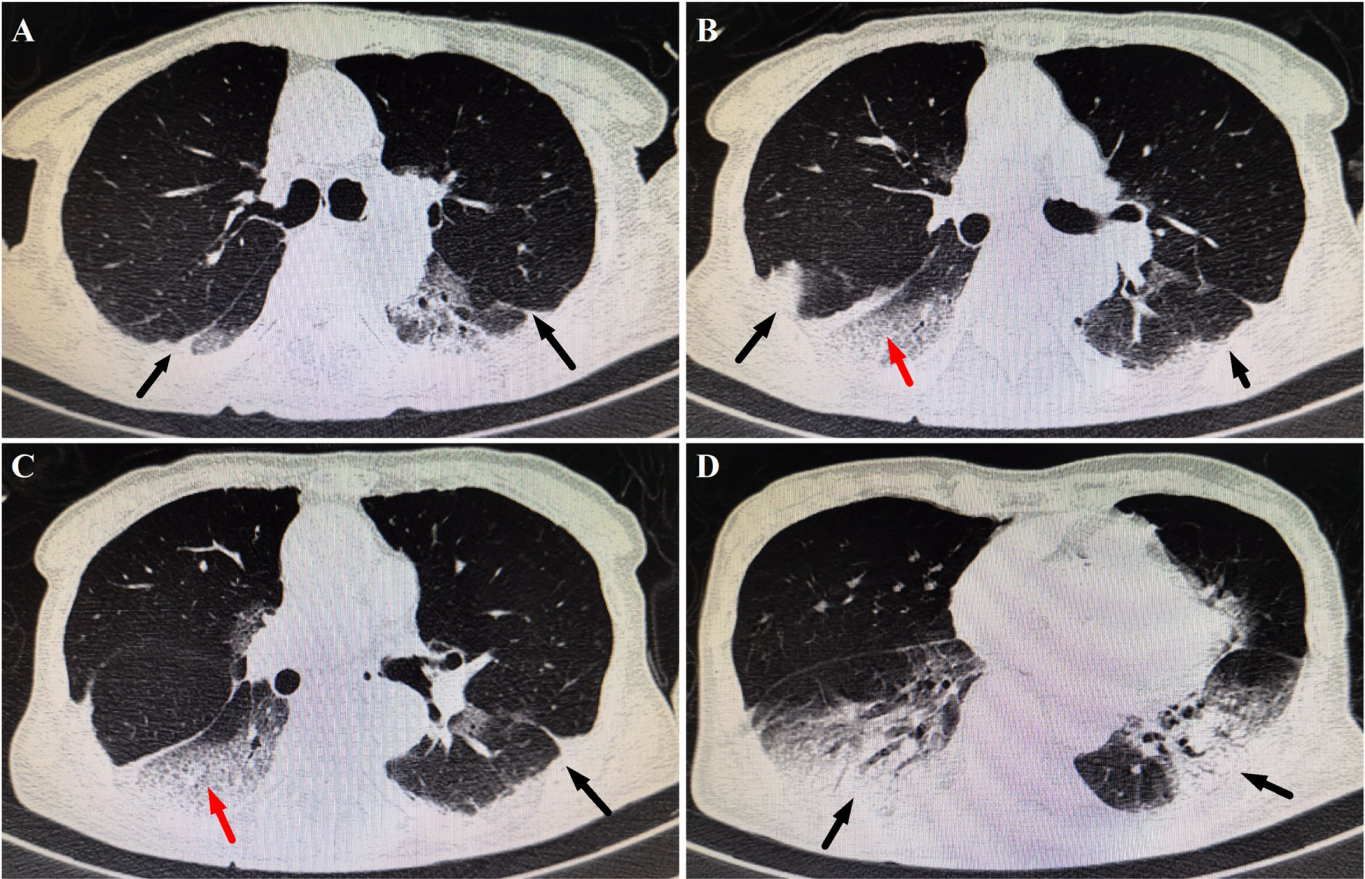
Selected images from chest computed tomography scans at different levels on hospital day 9. Subpleural multiple consolidations (black arrows) were noted to increase progressively, while crazy paving pattern decreased (red arrows), in comparison with the radiographic lesions on hospital day 5

The respiratory symptoms of the patient were gradually relieved. The repeat chest CT on hospital day 9 reported no exacerbations of lesions (Fig. 2). The patient’s oxygen saturation was stable on a nasal cannula at 2 L/min of supplemental oxygen. However, passage of dark red stools was present on hospital day 17. Laboratory results showed normocytic anemia (hemoglobin 8.8 g/dL), in the absence of coagulation dysfunction. Noticeably, the repeat rRT-PCR for SARS-CoV-2 testing was negative in respiratory specimens, but positive in stool specimens. The serum testing for tumor markers were all within normal ranges. Discouragingly, the site and causes of GIB weren’t well-established by the enhanced abdominal CT and CT angiography. Fasting and parenteral nutrition formulations were implemented. Intravenous resuscitation was started via 0.9% sodium chloride combined with 6 units of red blood cells within 48-h time period. Octreotide, hemocoagulase, and esomeprazole were administered for achieving hemostasis. However, refractory hematochezia was uncontrolled. The patient developed altered mental status, tachycardia (135 beats/min) and hypotension (62/28 mmHg). The hemoglobin progressively declined to 4.0 g/dL. Massive transfusions were administered, including red blood cells, 0.9% sodium chloride, and hydroxyethyl starch, combined with continuously intravenous infusion of dopamine. Unfortunately, on hospital day 21, despite aggressive resuscitation, the patient rapidly deteriorated, suffered from cardiac arrest, and expired, before interventional radiology and endoscopy were available. The family refused to autopsy.

## Discussion and conclusions

We presented a critically ill patient with COVID-19 who progressed rapidly with ARDS, and ultimately died due to massive GIB even after improvement of respiratory status. This case report highlighted the importance of early identification and prompt management to block the progression of the disease in the high-risk population, as well as the possibility of enteric involvement and faecal transmission.

Considering the rapidly progressive respiratory failure following initial therapy, chest CT was arranged. It was believed that chest CT imaging features seemed to be nonspecific and non-diagnostic. However, if the patient didn’t get improvement in symptoms or even worsened, progressive radiographic deterioration on CT scans developed, quickly evolving from focal unilateral to diffuse bilateral ground-glass opacities that progressed to or co-existed with consolidations within 1 ~ 3 weeks after symptom onset. At this stage, 11% of cases died [9]. Importantly, it was noted that multiple, patchy or large patches of consolidation associated with crazy paving pattern in both lungs (especially in the middle and lower lobes) were more commonly detected in elderly or severe condition patients. These radiographic appearance was similar to the positive CT findings in the present study. Subsequently, the following treatment regimens were implemented in this case. First, a more aggressive antibiotic treatment was administered based on the indicators of bacterial co-infection, including a delayed onset of dyspnea on illness day 20 relative to a median of 8 days from onset as reported [10], progressive elevation of leukocyte/CRP/PCT, and newly development of rales and fever. Second, it was observed that SARS-CoV-2 could induce cytokine storm, particularly in severely ill ones [10, 11]. In this context, methylprednisolone was administered in our patient according to the severity of the disease. Third, given its efficacy in preventing the disease deterioration, and shortening the length of intensive supportive care in critically ill patients [12, 13], IVIG was considered as an adjunctive drug. Finally, according to its capacity of repressing replication of virus as well as the anti-inflammatory and immunoregulatory effects, Chinese herbs were used in combination with conventional medicines [14]. The respiratory status of this patient resolved after administration of these above drugs, therefore, we speculated timely use of empirical antibiotics targeting the potential infection combined with complementary and supportive therapy might have beneficial effect in blocking the disease progression of COVID-19, especially for the severe or critically ill patients with high-risk factors.

It was reported that the critically ill patients were more likely to manifest digestive symptoms in comparison with non-severe patients, some of who merely presented with digestive symptoms, even in the absence of respiratory symptoms [15]. In this case, the clinical status of this patient deteriorated even after respiratory condition continued to improve. Hence, the cause of the patient’s death was considered to be strongly correlated with hemorrhagic shock owing to acute massive GIB rather than ongoing pneumonia. There have been some reports suggesting that patients treated with angiotensin receptor blockers may theoretically have increased numbers of ACE2 receptors, making them more susceptible to be infected with SARS-CoV-2 and probably higher risk for severe COVID-19 disease [16]. As our patient was taking candesartan daily as part of his regimen for hypertension, this may explain the severe condition in this case.

Endoscopic evaluation is generally undergone in hospitalized patients with GIB for the purpose of diagnostic and therapeutic purposes. Nevertheless, in view of the extremely high risk of virus transmission via aerosol during endoscopic procedures and limited personal protective equipment, endoscopy was ultimately not performed. This made it challenging to trace whether the GIB was primary or secondary outcomes of SARS-CoV-2 infection in this patient. Nevertheless, considering that the onset of digestive symptoms occurred after improvement of respiratory status and discontinuation of methylprednisolone, the possibility that the GIB secondary to hypoxemia and stress-induced ulcers might be minimal. More importantly, the rRT-PCR-positive results for SARS-CoV-2 in the repeat stool specimens should heighten the suspicion of direct viral invasion, conferring GIB subsequently. Given a prominently delayed presence of the positive faecal test (reported 2 ~ 5 days), whether the patient suffered from a new episode of viral attack targeting digestive system remained unclear. It was found that SARS-CoV-2 RNA in stool specimens might remain positive longer even after the clearance of the virus in the respiratory tract [17], suggesting that the virus survived longer in the gastrointestinal tract than in the respiratory tract. Therefore, a more thorough protocol for preventing cross-infection through faecal-oral transmission should be implemented in the process of patient care and infection control.

## Data Availability

The datasets used and/or analyzed during the current study are available from the corresponding author on reasonable request.

## Abbreviations

COVID-19: Coronavirus disease 2019
GIB: Gastrointestinal bleeding
rRT-PCR: Real-time reverse-transcriptase-polymerase chain reaction
CRP: C-reactive protein
PCT: Procalcitonin
CT: Computed tomography
HFNO: High flow nasal oxygen
IVIG: Intravenous immunoglobulin
